# Combination of computed tomography imaging-based radiomics and clinicopathological characteristics for predicting the clinical benefits of immune checkpoint inhibitors in lung cancer

**DOI:** 10.1186/s12931-021-01780-2

**Published:** 2021-06-28

**Authors:** Bin Yang, Li Zhou, Jing Zhong, Tangfeng Lv, Ang Li, Lu Ma, Jian Zhong, Saisai Yin, Litang Huang, Changsheng Zhou, Xinyu Li, Ying Qian Ge, Xinwei Tao, Longjiang Zhang, Yong Son, Guangming Lu

**Affiliations:** 1 Department of Medical Imaging, Affiliated Jinling Hospital, Medical School of Nanjing University, Nanjing, 210002 China; 2 Department of Respiratory and Critical Care Medicine, Affiliated Jinling Hospital, Medical School of Nanjing University, Sch Med, Nanjing, 210002 Jiangsu China; 3 Department of Respiratory and Critical Care Medicine, Affiliated Jinling Hospital, Southeast University, Sch Med, Nanjing, 210002 Nanjing China; 4 Department of Medical Imaging, Affiliated Jinling Hospital, Nanjing Medical University, Nanjing, 210002 China; 5 Siemens Healthineers Ltd., Shanghai, 200000 China

**Keywords:** Non-small cell lung cancer, Radiomics, Machine learning, Immune checkpoint inhibitors, Survival outcome

## Abstract

**Background:**

In this study, we tested whether a combination of radiomic features extracted from baseline pre-immunotherapy computed tomography (CT) images and clinicopathological characteristics could be used as novel noninvasive biomarkers for predicting the clinical benefits of non-small cell lung cancer (NSCLC) patients treated with immune checkpoint inhibitors (ICIs).

**Methods:**

The data from 92 consecutive patients with lung cancer who had been treated with ICIs were retrospectively analyzed. In total, 88 radiomic features were selected from the pretreatment CT images for the construction of a random forest model. Radiomics model 1 was constructed based on the Rad-score. Using multivariate logistic regression analysis, the Rad-score and significant predictors were integrated into a single predictive model (radiomics nomogram model 1) to predict the durable clinical benefit (DCB) of ICIs. Radiomics model 2 was developed based on the same Rad-score as radiomics model 1.Using multivariate Cox proportional hazards regression analysis, the Rad-score, and independent risk factors, radiomics nomogram model 2 was constructed to predict the progression-free survival (PFS).

**Results:**

The models successfully predicted the patients who would benefit from ICIs. For radiomics model 1, the area under the receiver operating characteristic curve values for the training and validation cohorts were 0.848 and 0.795, respectively, whereas for radiomics nomogram model 1, the values were 0.902 and 0.877, respectively. For the PFS prediction, the Harrell’s concordance indexes for the training and validation cohorts were 0.717 and 0.760, respectively, using radiomics model 2, whereas they were 0.749 and 0.791, respectively, using radiomics nomogram model 2.

**Conclusions:**

CT-based radiomic features and clinicopathological factors can be used prior to the initiation of immunotherapy for identifying NSCLC patients who are the most likely to benefit from the therapy. This could guide the individualized treatment strategy for advanced NSCLC.

## Background

According to the latest epidemiological data, lung cancer is the most common malignant cancer and has the highest morbidity and mortality rates worldwide [1]. Non-small cell lung cancer (NSCLC) accounts for more than 85% of the cases of lung cancer [2]. Despite significant advances in cancer diagnosis and treatment techniques, the long-term survival rate of patients with lung tumor is only 10–15%, and once metastasis occurs, the 5-year survival rate is lower than 5% [3]. Therefore, enhancing the efficacy of treatments is essential for improving the overall prognosis of patients with advanced NSCLC.

Although cytotoxic therapy remains an important player in systemic treatment, many chemotherapy regimens are associated with significant toxic effects [4].Compared with traditional cytotoxic agents, tyrosine kinase inhibitors (TKIs) have led to more favorable outcomes and have, thus, become the first-line treatment for patients with actionable driver mutations [5]. However, there are still many patients who do not have genetic mutations [6], who might benefit from immune checkpoint inhibitors (ICIs), particularly antibodies targeting the programmed cell death-1 (PD1)–programmed cell death ligand-1 (PD-L1) axis, which can restore the antitumor immune response of the body by blocking the immune suppression signals between the tumor and T cells [7]. A total of four ICIs have been approved by the Food and Drug Administration (FDA) for the treatment of lung cancer, including anti-PD1 antibodies (nivolumab and pembrolizumab) and anti-PD-L1 antibodies (atezolizumab and durvalumab) [8]. Intriguingly, the use of ICIs has shifted from the advanced stage to earlier stages of lung cancer in an attempt to reach the ultimate goal of curing the disease completely [9]. Despite the substantial progress made in immunotherapy research and development, only 20% of patients could derive benefits from ICIs [10]. Currently, PD-L1 expression and microsatellite instability/mismatch repair deficiency are approved for clinical use as predictive biomarkers of the response of tumors to ICIs [11]. However, multicohort studies, such as CheckMate 026, have shown that although a high tumor mutation burden is positively correlated with the efficacy of ICIs [12], this parameter is not a perfect biomarker for the selection of patients for ICI therapy [13]. Hence, there is an important and urgent need to identify and develop predictive biomarkers for immunotherapy. Moreover, an increasing number of studies have pointed out that combining different biomarkers to reduce the assumptive risk associated with each one can improve the performance of the prediction [14].

Radiomics is a noninvasive method with diagnostic, prognostic, and predictive value, which involves the conversion of image data into high-throughput image feature data, that can then be mined and used to describe the intensity, shape, and texture of tumors, and to quantify the heterogeneity of time and space of the tumor tissue [15–17]. The technique effectively transforms images into a high-dimensional recognizable feature space and uses statistical and/or machine learning methods to select the most valuable radiomic features for analyzing clinical information. The ultimate goal is to construct a model with diagnostic, prognostic, or predictive value that will provide valuable information for the establishment of an accurate individualized diagnosis and treatment strategy [18–20]. The nomogram is a graphical diagram that is easy to interpret and contains different kinds of predictors. In recent years, it has become an important tool for cancer research experts [21, 22]. In several studies, radiomics-based models may have a predictive and prognostic value in different kinds of cancers. When radiomic features are combined with clinicopathological factors, the model accuracy may further increase [23–25]. However, the predictive performance of the current prognostic prediction models are generally not very good [26–28].

Therefore, this study aims to construct radiomics nomogram models based on CT radiomic features and clinicopathological characteristics to predict the tumor response after ICIs and prognosis of patients with NSCLC.

## Methods

### Patients

**Fig. 1:**
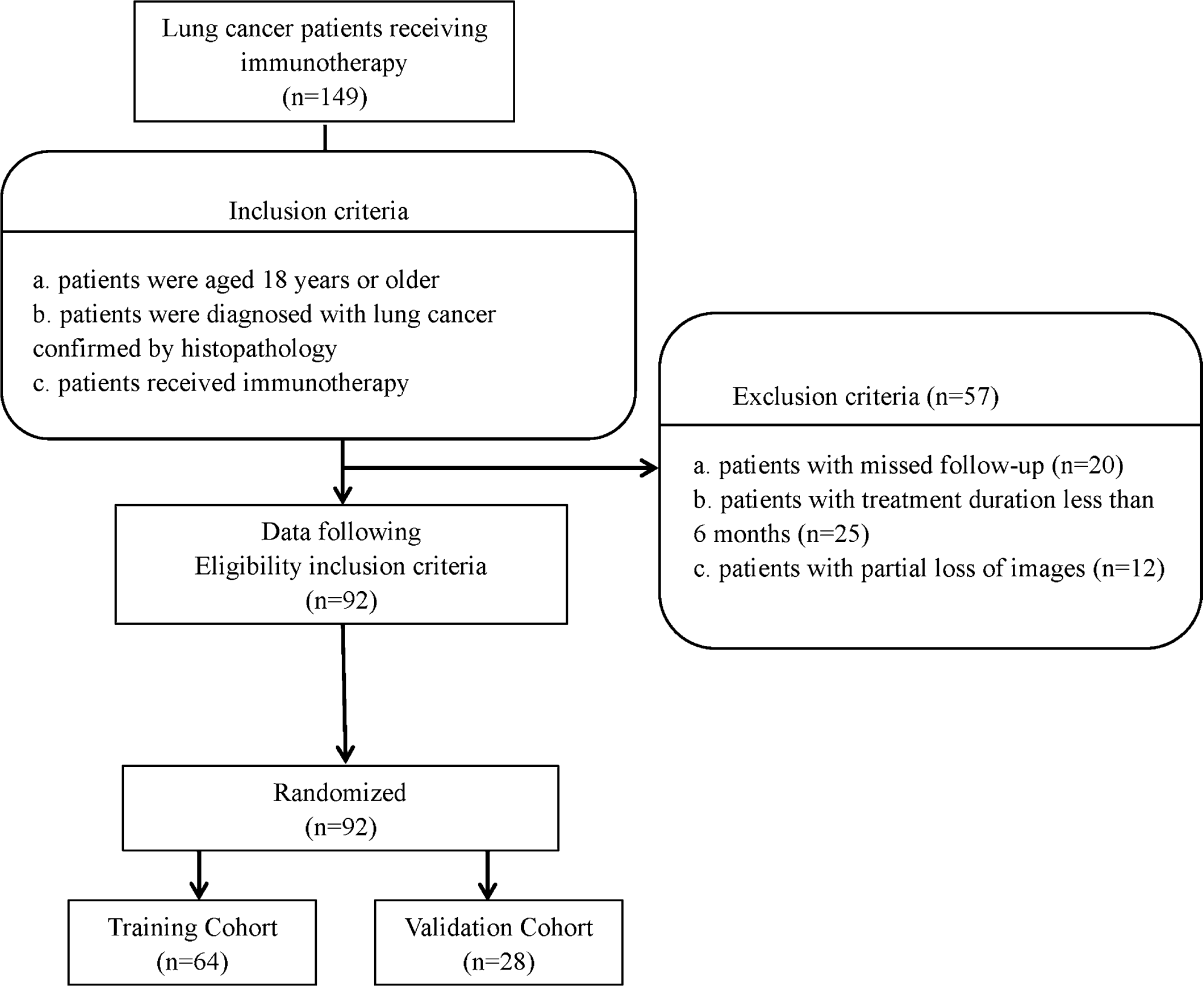
Flow diagram of the enrollment of patients with non-small cell lung cancer

We collected data from 149 patients with lung cancer at the Affiliated Jinling Hospital, Medical School of Nanjing University, between June 2015 and December 2019. The institutional review board of the Affiliated Jinling Hospital, Medical School of Nanjing University, approved this retrospective study and waived the informed consent requirements from the patients. However, consent was obtained from the patients upon publishing their images and clinical information. The inclusion criteria were the following: patients of 18 years of age or older; a diagnosis of lung cancer, as confirmed using histopathology, according to the eighth edition of the American Joint Commission on Cancer TNM classification and staging system; and patients receiving immunotherapy. The exclusion criteria were as follows: patients who missed the follow-up (n = 20); patients with a treatment duration of less than 6 months before progressive disease (PD) (n = 25); and patients with partial loss of images (n = 12). In total, 92 patients met the inclusion criteria and were randomly divided at a 7:3 ratio into the training cohort (n = 64) and the validation cohort (n = 28) (Fig. 1). For each patient, the baseline clinicopathological characteristics were obtained from their medical records, including the age, sex, smoking status, cancer family history, histological subtype, TNM classification and staging, blood cell counts (platelets, white blood cells, neutrophils, lymphocytes, and monocytes), levels of thyroid transcription factor 1 (TTF-1), Ki-67, C-reactive protein (CRP), carcinoembryonic antigen, and neuron-specific enolase, PD-L1 expression, line of therapy, and immunotherapy regimen. The time from the beginning of immunotherapy to the date of disease progression was defined as progression-free survival (PFS). The endpoint of this study was the clinical benefit of immunotherapy, which was defined as either a durable clinical benefit (DCB: complete response, partial response (PR), or stable disease (SD) lasting > 6 months) or no durable clinical benefit (NDB: PD or SD that lasted ≤ 6 months).

### Image acquisition

All patients underwent non-enhanced CT imaging of the lungs using one of three multidetector row CT systems (SOMATOM Definition Flash, SOMATOM Emotion, or SOMATOM Perspective, all from Siemens Healthineers AG, Erlangen, Germany). After removing any metallic foreign bodies from the chest area, the patient was placed in the supine position with both hands raised. The CT scan was conducted using the spiral scanning mode and ranged from the thoracic entrance to the underlying layer of the lung, with a single breath-hold scan at the end of inspiration. The following CT acquisition parameters were used: 120 or 130 kVp; 160 mAs; detector collimation: 6 × 1.25 mm, 64 × 0.625 mm, or 64 × 0.6 mm; rotation time: 0.5 or 0.8 s; matrix size: 512 × 512; field of view: 350 × 350 mm). Each patient was subjected to a whole-lung scan, and the CT image retrieved from the picture archiving and communication system was reconstructed using a standard kernel. The slice thickness of the CT images ranged from 1 to 1.25 mm.

### Image segmentation and radiomic feature extraction

**Fig. 2:**
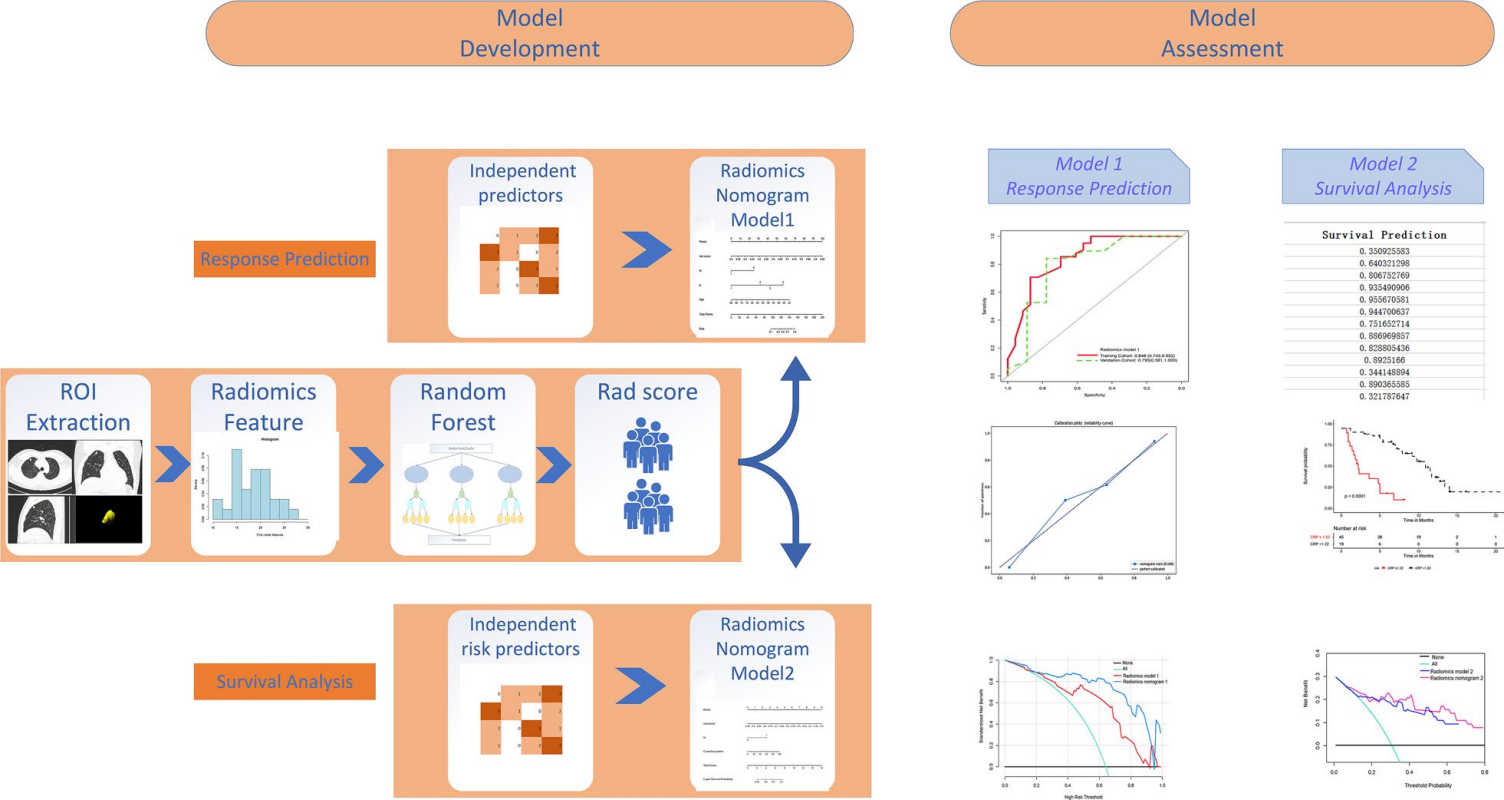
Workflow for developing the radiomics nomogram models. CT image segmentation was performed using manual semiautomatic segmentation using radiomics prototype software (Radiomics, Frontier, Siemens). The radiomic features from the volumes of interest were then computed using the CT images on the prototype. A predictive model was constructed on the basis of the CT-derived radiomic features using the random forest (RF) method to output a radiomics score (Rad-score) for each patient. The Rad-score was combined with significant clinicopathological factors for multivariate logistic regression analysis to develop radiomics nomogram model 1 to predict the durable clinical benefit (DCB). Radiomics nomogram model 2 was established to predict the progression-free survival (PFS) and was developed via multivariate logistic regression analysis of the Rad-score and significant risk factors combined

This study followed and adhered to the Image Biomarker Standardization Initiative (IBSI) guidelines [29] and the radiomics prototype software program used (Radiomics, Frontier, Siemens) was IBSI-compliant. A volume of interest was drawn semiautomatically around the tumor by a chest radiologist (Y.B., 9 years of experience) and was confirmed by another chest radiologist (Z.J., 15 years of experience). Both radiologists were blinded to the clinical information of the patients. First, we imported the CT images into Radiomics prototype software. In the segmentation module of Radiomics, a few segmentation tools are available for the semiautomatic delineation of the tumor in three dimensions. The segmentation is semiautomatically produced by drawing a line across the boundary of the tumor. Then, using an automatic algorithm, the tool finds neighboring voxels with the same gray level in the three-dimensional (3D) space, generating random walker-based lesion segmentation for solid and subsolid lung lesions [30]. If the segmentation was incorrect, the operators could correct it manually in the 3D domain using the Radiomics prototype. As a result, a total of 110 features (viz., 18 first-order, 75 texture, and 17 size and shape features) were extracted from the CT images using Radiomics. To test the intraclass reproducibility, the data from 25 randomly selected patients were segmented twice by one radiologist (Y.B.) within a 1-month period. To test the interclass reproducibility, the same 25 sets of data were segmented by two radiologists (Y.B. and Z.J.). Spearman correlation analysis was used to assess the differences between the features generated at different times and by different radiologists, as well as between the twice-generated features by the same radiologist. Interclass and intraclass correlation coefficients (ICCs) were used to evaluate the intra- and inter-observer agreement of the feature extraction, in which an ICC value greater than 0.80 indicated a good agreement. As a result, 88 features were retained for further analysis (Fig. 2).

### Predictive model construction and model testing

**Fig. 3:**
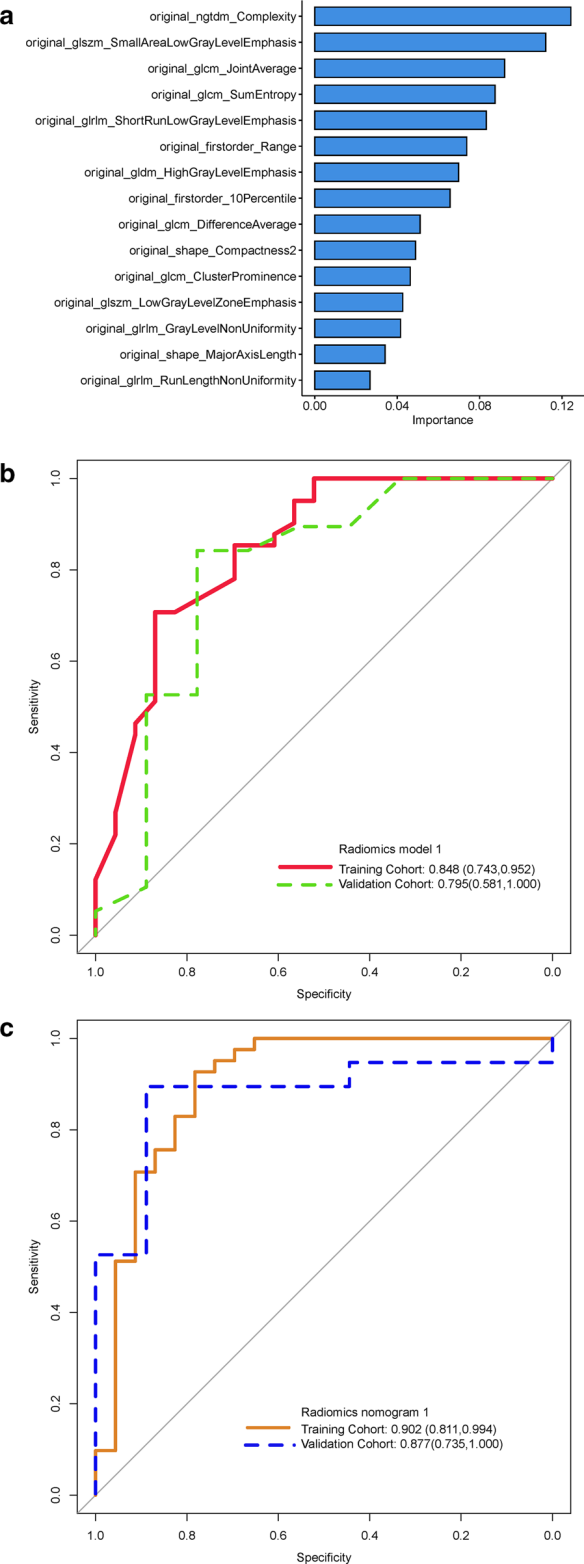
Receiver operating characteristic curves for the different models. **a** 15 important radiomic features were used to build the predictive models. **b** Receiver operating characteristic curves showing the differences between the training cohort and the validation cohort in radiomics model 1. **c** Receiver operating characteristic curves showing the differences between the training cohort and the validation cohort in radiomics nomogram model 1

A model for predicting the clinical benefits of immunotherapy was constructed on the basis of the CT-derived radiomic features, using the random forest (RF) method. Patients with DCB were labeled as positive, and those with NDB, as negative. The RF algorithm has a comparably low tendency to overfit and is well suited for datasets with a large number of heterogeneous predictors and cluster-correlated observations; thus, it was adopted for the machine learning-based prediction model. The RF method was used to construct the prediction model because of its high variance bias trade-off capability. It is a classification algorithm consisting of many decision trees, in which each tree represents a weak classifier. A combination of trees can achieve an improved model performance. The split quality was measured according to the Gini impurity. Hyperparameter optimization was performed. The RF model was evaluated in an additional independent cohort study, for which potentially unseen test patients were randomly selected. The performance of the model was assessed based on its receiver operating characteristic (ROC) curve, area under the ROC curve (AUC), accuracy, sensitivity, and specificity. Finally, radiomics model 1 was established based on 15 important radiomic features (Fig. 3a). The forest consisted of five trees, and the maximum depth of the tree was set as 2. Three features were considered when looking for the best split. Ultimately, a radiomics score (Rad-score) was assigned to each patient.

### Clinicopathological factors plus radiomics model development and radiomics nomogram model construction

The clinicopathological factors were analyzed using univariate logistic regression analysis, in which the predictors with a *p* value lower than 0.10 were included to find those of significance. Through multivariate logistic regression analysis, the multimodal features, Rad-score, and significant predictors were then integrated into a single predictive model, whereupon radiomics nomogram model 1 was constructed for the training cohort. Calibration curves were plotted to assess the calibration of radiomics nomogram model 1. Decision curve analysis was conducted to determine the clinical usefulness of the radiomics model and radiomics nomogram model by quantifying the net benefits at different threshold probabilities for the training and validation cohorts.

### Clinicopathological factor analysis

The clinicopathological factors were analyzed using univariate Cox proportional hazards (CPH) regression analysis. Those factors with a *p* value lower than 0.10 and the Rad-score were then combined for multivariate CPH regression analysis to identify the independent risk factors, which were subsequently analyzed using the Kaplan–Meier curve and log-rank test. The final model was selected via backward stepwise elimination, with the Akaike information criterion as the stopping rule [31].

### Construction of the radiomics nomogram model 2

The same Rad-score was used to construct radiomics model 2. The multimodal features and parameters, including the Rad-score and independent risk factors, were integrated into a single predictive model using multivariate CPH regression analysis, upon which radiomics nomogram model 2 was developed for the training cohort. The prognostic abilities of the generated models were evaluated using the training cohort and validated using the validation cohort. The discrimination performance of the prognostic models was assessed using Harrell’s concordance index (C-index), which ranges from 0.5 (indicating a random distribution of the data) to 1.0 (indicating a perfect prediction of the observed survival information by the model). Calibration curves of the nomogram were subsequently drawn for the 2-year PFS of the patients. The calibration curves were used to determine the independent risk factors, as well as to indicate both the PFS probabilities predicted by the prognostic models and the observed probabilities.

### Statistical analysis

The differences in the clinicopathological factors between the training and validation datasets were assessed using the Mann–Whitney U test, for continuous variables, and the χ^2^ test, for categorized variables. The discrimination power of the models was measured based on the AUC and the C-index. Delong test was used to compare two AUCs, and the log-likelihood ratio was used to assess the increase in the predictive power of the C-index. The radiomics nomogram model 2 score was calculated and used to assess risk stratification. Survival curves were generated using the Kaplan–Meier method and compared using the two-sided log-rank test. The optimal cutoff point for continuous prognostic markers in the survival analysis was determined using X-tile software (version 3.6.1; Yale University School of Medicine, New Haven, CT, USA). A calibration curve was generated to demonstrate the goodness of the fit, which is a graphical representation of the relationship between the observed and the predicted survival. The Greenwood-Nam-D’Agostino (GND) method was applied to measure the statistical significance of the goodness-of-fit test results. The prediction error of the models, which was assessed using the “Boot632plus” split method by performing 100 iterations to calculate the estimates of the prediction error curves, was summarized as the integrated Brier score, which represents a valid measure of the overall model performance and can range from 0 (for a perfect model) to 0.25 (for a noninformative model with a 50% incidence on the outcome). The RF model was established using Python software (Python Scikit-learn package comprising Python version 3.7 and Scikit-learn version 0.21; http://scikit-learn.org/). The construction of radiomics nomogram model 2, the assessment of the model, and decision curve analysis (DCA) were performed using R software (version 3.4.4; http://www.r-project.org). All the codes are available at https://github.com/tomato0821 7/immune. All statistical tests were two-sided, with a significance level of 0.05.

## Results

### Clinicopathological characteristics of the patients

The baseline clinical characteristics of the patients in the training and validation cohorts are listed in Table 1. There were no significant differences in sex, age, smoking status, family history, histology type, cancer stage, TTF-1, and Ki-67 (*p* = 0.070–1.000) between the two cohorts.

**Table 1:** Demographic and clinical characteristics of the patients

Characteristic	Training cohort	Validation cohort	*p*-value
	(n = 64)	(n = 28)	
Sex, n. (%)			0.086
Female	14 (21.88)	11 (39.29)	
Male	50 (78.12)	17(60.71)	
Age(years), mean (SD)	20.44 (8.98)	20.04 (8.99)	0.844
Smoking status, n. (%)			0.656
No	34 (53.10)	17 (60.7)	
Yes	30 (46.90)	11 (39.3)	
Family history, n. (%)			0.754
No	62 (96.90)	26 (92.9)	
Yes	2 (3.10)	2 (7.1)	
TTF-1, n. (%)			0.070
Negative	42 (65.60)	12 (42.9)	
Positive	22 (34.40)	16 (57.1)	
Ki-67, n. (%)			0.560
Low expression	33 (51.60)	17 (60.7)	
High expression	31 (48.40)	11 (39.3)	
Histologic type, n. (%)			0.120
Adenocarcinoma	32 (50.00)	20 (71.43)	
Squamous cell carcinoma	28 (43.80)	6 (21.43)	
NOS	4 (6.20)	2 (7.14)	
Stage, n. (%)			0.257
Ш	23 (35.90)	6 (21.40)	
IV	41 (64.10)	22 (78.60)	
T stage, n. (%)			0.842
0	1 ( 1.60)	0 ( 0.00)	
1	8 (12.50)	3 (10.70)	
2	20 (31.20)	11 (39.30)	
3	9 (14.10)	5 (17.90)	
4	26 (40.60)	9 (32.10)	
N stage, n. (%)			0.821
0	6 (9.38)	3 (10.70)	
1	5 (7.81)	1 (3.60)	
2	25 (39.06)	13 (46.40)	
3	28 (43.75)	11 (39.30)	
M stage, n. (%)			0.176
0	22 (34.40)	5 (17.90)	
1	42 (65.60)	23 (82.10)	
Lymph node metastasis, n. (%)			0.880
No	9 (14.10)	5 (17.90)	
Yes	55 (85.90)	23 (82.10)	
Intrapulmonary metastasis, n. (%)			0.466
No	39 (60.90)	20 (71.40)	
Yes	25 (39.10)	8 (28.60)	
Brain metastasis, n. (%)			0.330
No	55 (85.90)	21 (75.00)	
Yes	9 (14.10)	7 (25.00)	
Liver metastasis, n. (%)			1.000
No	59 (92.20)	026 (92.90)	
Yes	5 ( 7.80)	2 (7.10)	
Bone metastasis n. (%)			1.000
No	44 (68.80)	019 (67.90)	
Yes	20 (31.20)	9 (32.10)	
Pleural metastasis n. (%)			0.073
No	54 (84.38)	19 (67.86)	
Yes	10 (15.62)	9 (32.14)	
White blood cell, (median [IQR])	6.40 [4.47, 7.78]	6.15 [5.00, 8.33]	0.333
Neutrophil, (median [IQR])	69.05 [62.65,73.78]	65.20 [58.50, 71.08]	0.203
Monocyte, (median [IQR])	8.65 [6.83, 11.03]	6.70 [5.27, 9.45]	0.952
CRP, (median [IQR])	18.10 [5.05, 19.85]	12.00 [1.17, 18.83]	0.330
CEA, (median [IQR])	13.60 [3.88, 84.30]	11.30 [2.80, 84.30]	0.568
NSE, (median [IQR])	14.25 [10.35, 19.10]	15.05 [12.00, 19.10]	0.513
PD-L1 expression, n. (%)			0.9276
< 1%	30 (46.88)	12 (42.86)	
≥ 1%	12 (18.75)	6 (21.43)	
Unknown	22 (34.37)	10 (35.71)	
Therapy line, n. (%)			0.7251
1st	23 (35.94)	9 (32.14)	
≥ 2nd	41 (64.06)	19 (67.86)	
Immunotherapy regimen, n. (%)			0.9366
PD-1 inhibitors	36 (56.25)	16 (57.14)	
PD-1 inhibitors + chemotherapy	28 (43.75)	12 (42.86)	

*CRP* C-reactive protein, *CEA* carcinoembryonic antigen, *NSE* neuron-specific enolase, *NOS* not otherwise specified, four are adenosquamous carcinoma and two are small cell lung cancer

### Prognostic model performance and development of the radiomics nomogram model 1

**Fig. 4:**
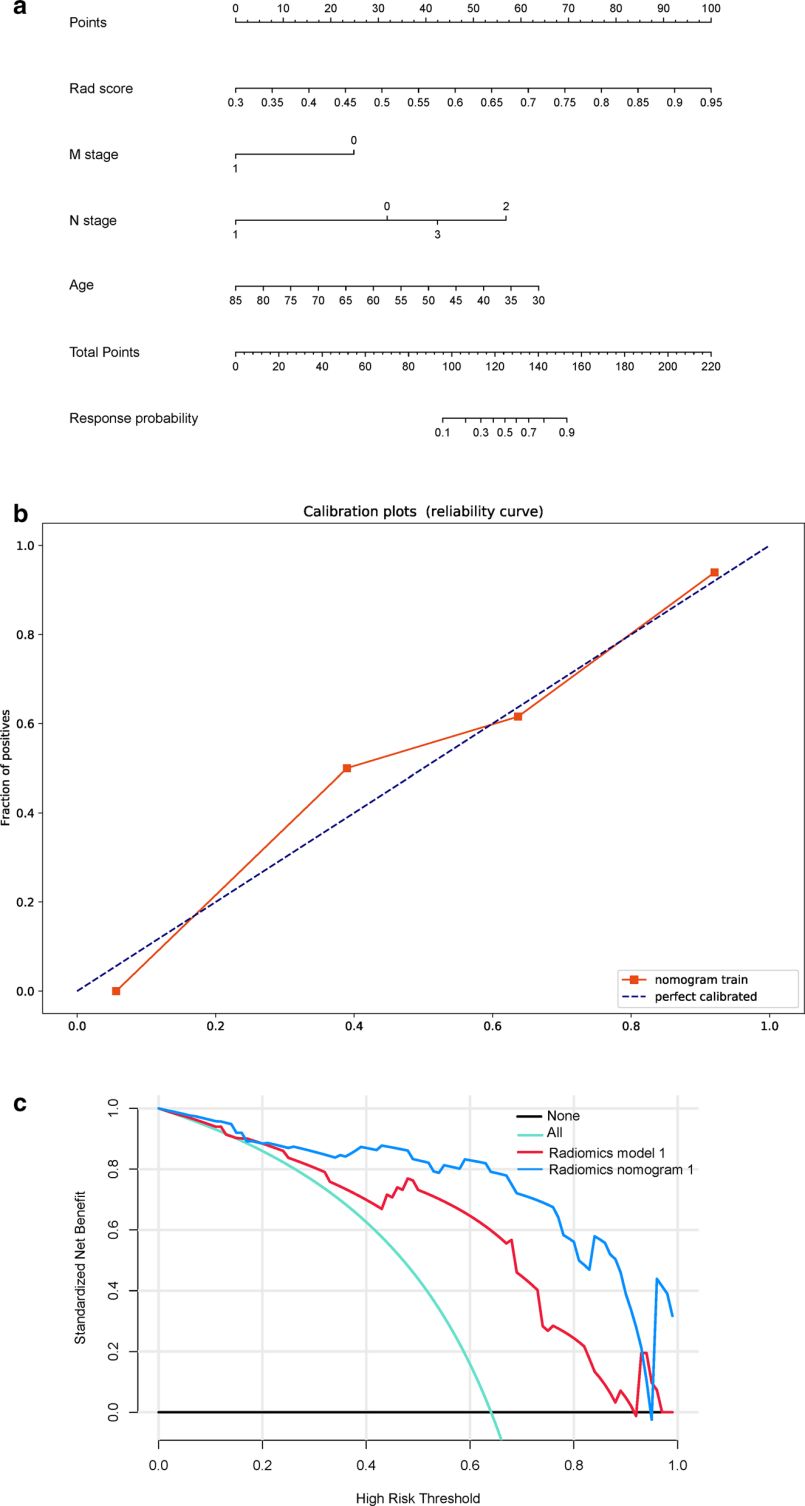
Development of radiomics nomogram model 1. **a** Nomogram based on independent predictors (Rad-score, Age, N stage and M stage). **b** Calibration curves of the nomogram in the training cohort. The horizontal axis is the predicted incidence of the durable clinical benefit (DCB), whereas the vertical axis is the observed incidence of the DCB. The dotted line on the diagonal is the reference line at which the predicted value is equal to the actual value. The orange line is the calibration curve. **c** Decision curve analysis for each model. The y-axis measures the net benefit, which was calculated using true-positive and false-positive results. Radiomics nomogram model 1 had the highest net benefit among all positive predictions (line labeled “All”), all negative predictions (line labeled “None”), and models (line labeled “radiomics model 1”) at the threshold from 0.1 to 0.9

In total, 15 CT-derived radiomic features were used to build radiomics model 1. Logistic regression analysis had identified the Rad-score (OR, 1.270 × 10^5^), age (OR, 0.910), and stage M (OR, 0.153) as being independent predictors (Table 2). Multivariate logistic regression analysis of the Rad-score combined with the clinicopathological factors, was used to build the model, which was designated as radiomics nomogram model 1. After tenfold cross-validation, radiomics model 1 was found to have an AUC value of 0.848, a sensitivity value of 0.625, a specificity value of 0.906, an accuracy value of 0.766, a positive predictive value of 0.870, and a negative predictive value of 0.707 for the training cohort, whereas it had an AUC value of 0.795, a sensitivity value of 1.000, a specificity value of 0.704, an accuracy value of 0.714, a positive predictive value of 0.111, and a negative predictive value of 1.000 for the validation cohort (Fig. 3b). For radiomics nomogram model 1, the AUC value was 0.902, the sensitivity value was 0.857, the specificity value was 0.884, the accuracy value was 0.875, the positive predictive value was 0.783, and the negative predictive value was 0.927 for the training cohort, whereas the AUC value was 0.877, the sensitivity value was 0.800, the specificity value was 0.944, the accuracy value was 0.893, the positive predictive value was 0.889, and the negative predictive value was 0.895 for the validation cohort (Fig. 3c and Table 3). The independent predictors were presented as radiomics nomogram model 1 (Fig. 4a). The calibration curve for the probability of DCB and NDB in the training and validation cohorts demonstrated a good agreement between the predicted and actual values (Fig. 4b). The Brier scores also showed the good performance of the model, with values of 0.178 and 0.187 for the training and the validation cohorts, respectively. Decision curve analysis was performed to determine the clinical usefulness of radiomics nomogram model 1 by quantifying the net benefits at different threshold probabilities. The results showed that radiomics nomogram model 1 had a higher overall net benefit than radiomics model 1 across the majority of the range of reasonable threshold probabilities (Fig. 4c).

**Table 2:** Multivariable logistic regression analysis for nomogram model construction

	Odds ratio	95%CI		*P*
		Lower	Upper	
Rad score	1.27010^5^	3.832	3.41810^8^	< 0.001
Age	0.910	0.184	0.989	0.045
Stage n1	0.075	5.88010^–5^	4.0456	0.260
Stage n2	6.030	0.421	86.171	0.171
Stage n3	1.990	0.139	23.380	0.584
Stage M	0.153	0.022	0.777	0.035

*OR* odds ratio

**Table 3:** Predictive performance of the two models in the training and validation cohorts

	Radiomics model1		Radiomics nomogram model1	
	Training cohort	Validation cohort	Training cohort	Validation cohort
AUC (95%CI)	0.848 (0.743–0.952)	0.795 (0.581–1.000)	0.902 (0.811–0.994)	0.877 (0.735–1.000)
Accuracy (%)	0.766	0.714	0.875	0.893
Sensitivity (%)	0.625	1.000	0.857	0.800
Specificity (%)	0.906	0.704	0.884	0.944
Positive predictive value (%)	0.870	0.111	0.783	0.889
Negative predictive value (%)	0.707	1.000	0.927	0.895

### Clinicopathological factor analysis

**Fig. 5:**
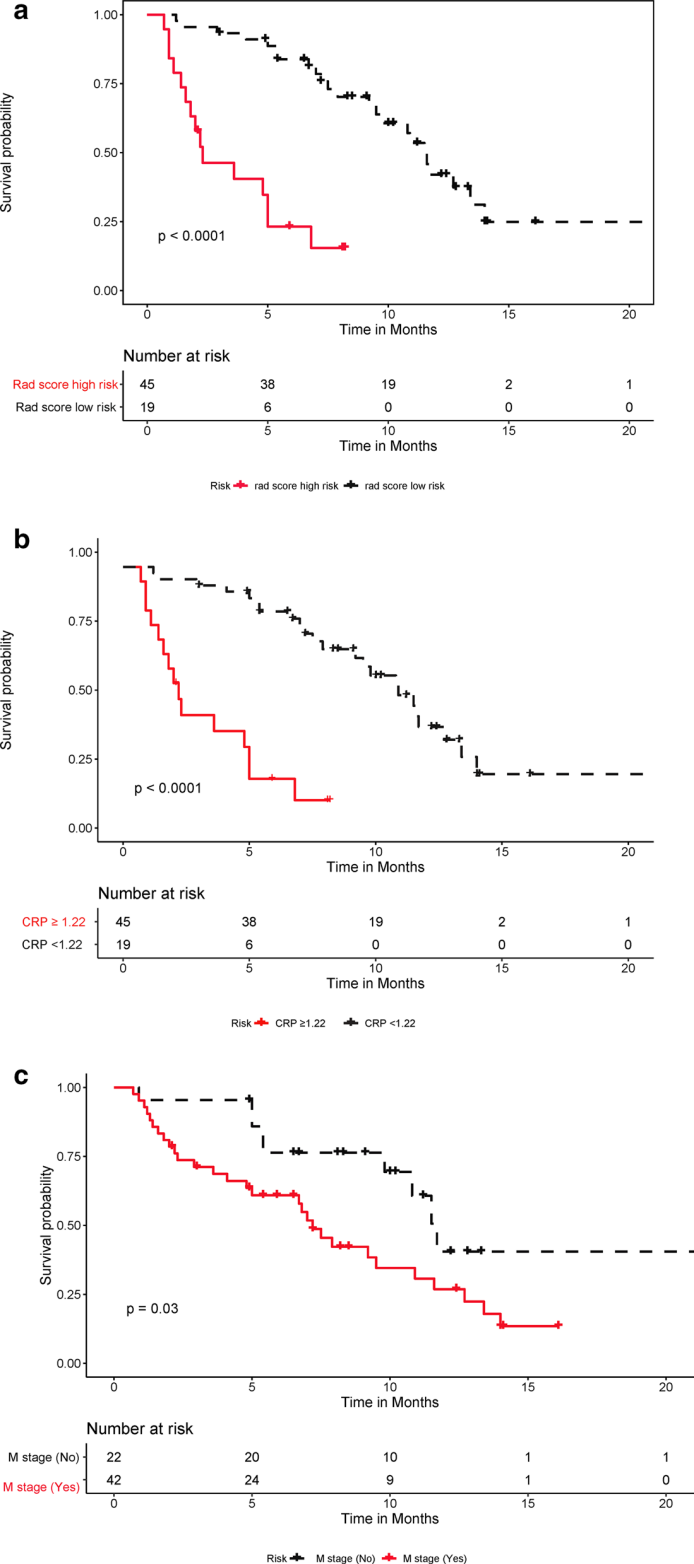
Predictive performances of the Rad-score, C-reactive protein (CRP) level, and M stage (**a**) Kaplan–Meier analysis of the Rad-score. The patients were stratified into high- and low-risk groups based on the Rad-score (A,* p* < 0.0001, log-rank test). **b** Kaplan–Meier analysis of the CRP level. The patients were stratified into high- and low-risk groups based on the CRP level (B,* p* < 0.0001, log-rank test). **c**. Kaplan–Meier analysis of M stage. The patients were stratified into high- and low-risk groups on the basis of M stage (C,* p* = 0.03, log-rank test)

The clinicopathological factors were analyzed using univariate CPH regression analysis to test the hazard ratio of each factor and to determine its significance in the probability of PFS. The factors with a *p* value lower than 0.10 were then combined for multivariate CPH regression analysis to identify the independent risk factors, whereupon the Rad-score (HR, 0.005)and M stage (HR, 2.449) were found to be the independent risk factors of the patients (Table 4). These risk factors were then analyzed using the Kaplan–Meier curve and log-rank test, with the latter in the cases of significant discrimination between the two groups. Figure 5a–c shows the PFS probability of the patients in the high-risk or low-risk cohorts.

**Table 4:** Multivariable Cox proportional hazards regression analysis for nomogram model construction

	HR	95%CI		*P*
		Lower	Upper	
Rad score	0.005	4.572*10^–3^	0.043	< 0.001
CRP	1.015	0.996	1.035	0.130
M stage	2.449	1.138	5.269	0.020

*HR* hazard ratio, *CRP* C-reactive protein

### Prognostic prediction model performance and development of radiomics nomogram model 2

**Fig. 6:**
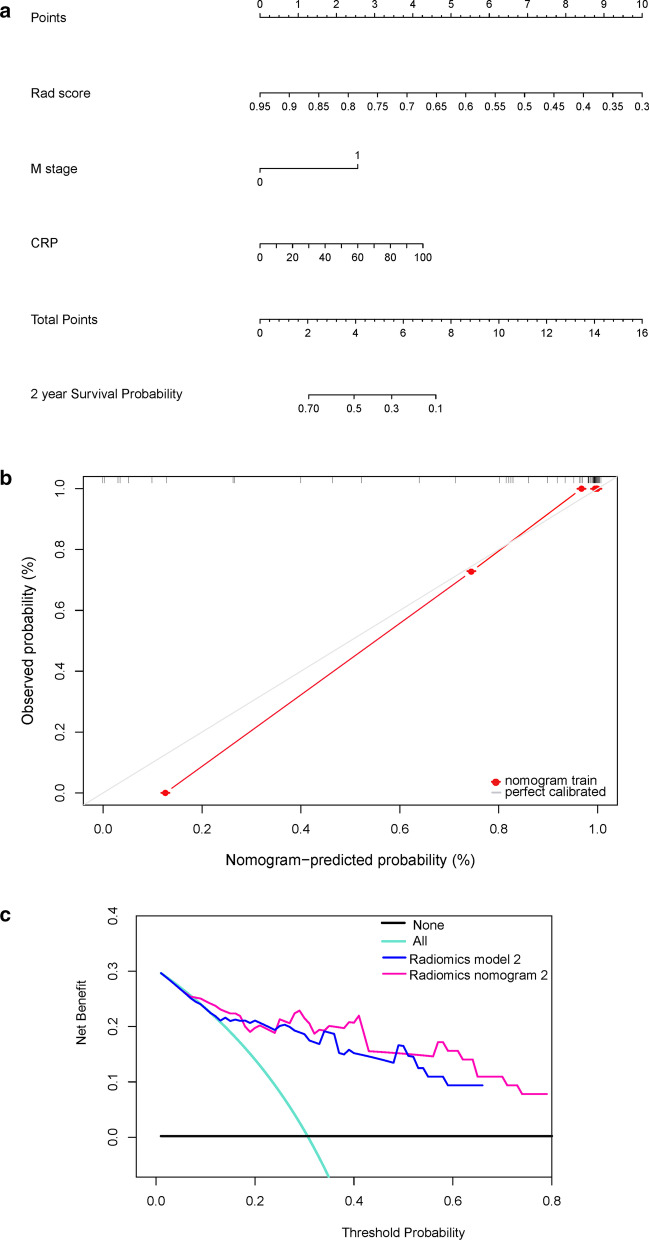
Development of radiomics nomogram model 2. **a** Nomogram based on independent risk factors (Rad-score, M stage, and C-reactive protein (CRP) level). **b** Calibration curves of the nomogram in the training cohort. The horizontal axis is the predicted incidence of progression-free survival (PFS), whereas the vertical axis is the observed incidence of PFS. The gray line on the diagonal is the reference line at which the predicted value is equal to the actual value. The red line is the calibration curve. **c** Decision curve analysis for each model. The y-axis measures the net benefit, which was calculated using true-positive and false-positive results. Radiomics nomogram model 2 had the highest net benefit among all positive predictions (line labeled “All”), all negative predictions (line labeled “None”), and models (line labeled “radiomics model 2”) at a threshold from 0.1 to 0.9

For radiomics model 2, the C-indexes were 0.717 and 0.760 for the training and validation cohorts, respectively, whereas for radiomics nomogram model 2, the values were 0.749 and 0.791, respectively (Table 5). The risk factors were presented as the nomogram (Fig. 6a). The calibration curve showed that the predicted probability was significantly close to the actual PFS of patients, with a *p* = 0.21 in the GND goodness-of-fit test (Fig. 6b). The Brier scores also showed the good performance of the model, with values of 0.187 and 0.209 for the training and test datasets, respectively. The decision curve analysis of the clinical usefulness of radiomics nomogram model 2 showed that the model had a higher overall net benefit than radiomics model 2 across the majority of the range of reasonable threshold probabilities (Fig. 6c).

**Table 5:** Harrell’s concordance indexes for the different modalities

Modalities	Training cohort (n = 64)		Validation cohort (n = 28)	
	C-index 95%CI	Brier score	C-index 95%CI	Brier score
Radiomics model2	0.717 (0.612, 0.822)	0.178	0.760 (0.574, 0.946)	0.187
Radiomics nomogram mode2	0.749 (0.643, 0.854)	0.187	0.791 (0.605, 0.978)	0.209

C-index, concordance index

## Discussion

In this study, an RF model based on the radiomic features of CT images and a radiomics model 1 based on the Rad-score were established, following which multivariate logistic regression analysis of the Rad-score, combined with the clinicopathological factors, was carried out to construct a radiomics nomogram model (radiomics nomogram model 1) for distinguishing patients who received DCB from those who received NDB after ICI treatment. Then, a second radiomics model (radiomics model 2) based on the Rad-score was established, following which multivariate CPH regression analysis of the Rad-score, combined with independent risk factors, was conducted to establish a second radiomics nomogram model (radiomics nomogram model 2) for predicting the PFS after immunotherapy. The ultimate goal is to use these models to identify patients who can benefit from immunotherapy, to provide guidance for individualized immunotherapy, and to establish a reference for the advancement of precision medicine.

Multivariate logistic regression analysis of the Rad-score and the significant clinicopathological factors combined showed that the Rad-score, age, and M stage were independent predictors, indicating that patients with a higher Rad-score, a younger age, and in the M0 stage can benefit from immunotherapy. The prediction models had good prediction performances. Radiomics model 1 had an AUC value of 0.848 for the training cohort, whereas it had an AUC value of 0.795 for the validation cohort. The independent predictors (Rad-score, age, N stage and M stage) were used to build radiomics nomogram model 1, whose performance was good, with an AUC value of 0.902 in the training cohort, and an AUC value of 0.877 in the validation cohort. Mu et al. [32], based on the radiomics of pretreatment ^18^F-FDG PET/CT images, predicted the clinical benefit of checkpoint blockade immunotherapy for patients with advanced NSCLC. Their results showed that the multiparametric radiomics signature could predict the DCB for patients, with AUC values of 0.86 (95%CI, 0.79–0.94), 0.83 (95%CI, 0.71–0.94), and 0.81 (95%CI, 0.68–0.92) for the training, retrospective test, and prospective test cohorts, respectively. Trebeschi et al. [33] performed an artificial intelligence-based characterization of each lesion on the pretreatment contrast-enhanced CT imaging data to develop and validate a noninvasive machine learning biomarker for distinguishing between anti-PD1 immunotherapy responding and non-responding patients with advanced melanoma and NSCLC. Their results showed that the biomarker achieved a significant performance for NSCLC lesions (AUC = 0.83, *p* < 0.001) and borderline significance for melanoma lymph nodes (AUC = 0.64, *p* = 0.05). After combining these lesion-wide predictions at the patient level, the immunotherapy response could be predicted with an AUC value of up to 0.76 for both cancer types (*p* < 0.001). Thus, the performance of our prediction model is more encouraging than that of previous studies, possibly due to the following reasons. First, it may be attributed to our implementation of a cross-validation approach and use of a performance-driven feature selection strategy and the RF algorithm for model training (which is overfitting-robust) to build a reliable model with higher performance. These strategies have been proposed in previous studies to achieve a better model performance [34, 35]. Additionally, independent predictors were selected to construct the models, and the combination of these clinical predictors may have improved the model performance.

Moreover, the same Rad-score was used to construct radiomics model 2, whose C-indexes were 0.717 and 0.760 for the training and validation cohorts, respectively. The Rad-score was combined with significant factors (*p* < 0.10) for multivariate CPH regression analysis to identify the independent risk factors, whereupon the Rad-score and M stage were identified as such for patients. It was demonstrated that disease progression after the receival of immunotherapy was more common in the patients with a lower Rad-score and in the M1 stage. Radiomics nomogram model 2 was established using the same three independent risk factors and the C-indexes were 0.749 and 0.791 for the training and validation cohorts, respectively. Our models had better predictive performance than that of a previous study [32].The independent predictors and independent risk factors were presented as nomograms, in which the calibration curves for the probability of response prediction or survival analysis demonstrated good agreement between the prediction and the observation. Moreover, the decision curve analysis showed that the radiomics nomogram models had a higher overall net benefit than the radiomics models across the majority of the reasonable threshold probabilities. This demonstrates that our models had the highest net benefit in guiding clinical decision-making. To our best knowledge, this is the first study to have used a noninvasive radiomics approach based on CT images and the clinicopathological characteristics to predict the efficacy of ICIs in Chinese patients with lung cancer.

Our study has some limitations; namely, the relatively small cohort size due to the relatively late date of approval of the ICIs (in 2018) by the China Food and Drug Administration; the use of only a single-center cohort; the retrospective nature of the data; and the lack of external validation, which may have introduced selection bias. However, we plan to rapidly expand the cohort size and to recruit multicenter cohorts to validate the models in the near future. Additionally, we did not conduct research on the overall survival but will include this in future studies.

## Conclusions

In conclusion, by combining CT image-based radiomics and clinicopathological factors, radiomics nomogram models were constructed for identifying patients with NSCLC who would most likely benefit from immunotherapy, resulting in a longer PFS. The good performance of the models suggests that they could be used to provide more precise guidance for the administration of immunotherapy to NSCLC patients.

## Data Availability

Datasets are available on request. The raw data supporting the conclusions of this manuscript will be made available by the authors, without undue reservation, to any qualified researcher. Requests to access the datasets should be directed to Dr. Guangming Lu (E-mail: cjr.luguangming@vip.163.com).

## Abbreviations

CI: Confidence interval
C-index: Harrell’s concordance index
NSCLC: Non-small cell lung cancer
TNM: Tumor-node-metastasis
TKIs: Tyrosine kinase inhibitors
ICIs: Immune checkpoint inhibitors
CRP: C-reactive protein
PFS: Progression-free survival
DCB: Durable clinical benefit
PR: Partial response
SD: Stable disease
NDB: No durable clinical benefit
ROC: Receiver operating characteristic
AUC: Area under the receiver operating characteristic curve
CPH: Cox proportional hazards
